# Characterization of indoor arenas through an anonymous survey

**DOI:** 10.1093/tas/txab198

**Published:** 2021-10-09

**Authors:** Staci McGill, Morgan Hayes, Kimberly Tumlin, Robert Coleman

**Affiliations:** 1 Department of Biosystems and Agricultural Engineering, University of Kentucky, 128 CE Barnhart Building, 1398 Nicholasville Rd, Lexington, KY40546, USA; 2 Department of Epidemiology, University of Kentucky, 720 Sports Center Drive, Lexington, KY 40506, USA; 3 Department of Animal and Food Sciences, University of Kentucky, 613 W.P. Garrigus Building, Lexington, KY 40546, USA

**Keywords:** equine, equine facilities, indoor air quality, indoor arenas

## Abstract

Equine farms are building both stables for the horses to live in and additional facilities to train and work horses (Kidd et al., 1997). For many of these farms, an outdoor arena that has an all-weather footing is the first working facility built. During inclement weather the ability to train in the outdoor arenas is inhibited, which in turn means the trainers, riders, and farms lose income as money is only made when horses are working, training, and competing. Indoor arenas allow for horses to continue to be worked no matter the weather conditions. The equine industry contributes a total of $122 billion dollars a year to the United States’ economy. The expenditures to build and maintain these arenas the horses utilize for training and work are a portion of the equine economic contribution (American Horse Council Foundation, 2018). During the summer of 2018, an anonymous online survey was conducted to begin to characterize indoor arenas. Owners, managers, and riders were questioned on a variety of topics including arena construction and design, arena usage, footing type, maintenance practices, environmental concerns, and potential health issues experienced within the facilities. Respondents in the study defined indoor arenas differently depending on geographic region, however most definitions included a roof, some enclosure, and footing in order to work the horses. In addition, of the 335 respondents of the survey, 71% or 239 respondents reported having concerns about the environment within the indoor arena. The three main concerns are dust, moisture, and lack of air movement. Overall, the survey begins to build our understanding regarding these facilities and provides the framework to continue research in the future.

## INTRODUCTION

The equine industry contributes a total of $122 billion dollars a year to the United States’ economy which indicates that significant money is spent on horse farms including infrastructure the horses utilize (American Horse Council Foundation, 2018). Not only do farms build stables for the horses to live in, but also facilities required to train and work horses (Kidd et al., 1997). Many of these farms have outdoor arenas, but inclement weather can inhibit the ability to train in the outdoor arenas so often indoor arenas are built. An indoor arena affords trainers and riders the ability to keep their horses exercising and training even in inclement weather. These facilities potentially receive high amount of use and yet there is very little information on common characteristics, environmental control features, or potential health hazards within these spaces.

Publications of equine facility design recommendations and considerations has been centered in extension publications and, primarily, focus on the barns or stables where horses live. Clemson University (1991), the Alberta Agriculture, Food, and Rural Development (1997), Pennsylvania State University (2002), and Iowa State University (2005) are the most notable institutes to publish information on the best design practices. Clemson University’s booklet focuses on different plans for stables based on the number of stalls required (“Horse Barns and Equipment,” 1991). In contrast, the Alberta Agriculture, Food and Rural Development discussed many aspects of the horse facilities including barn design, fencing considerations, feed containers, and arenas (Kidd et al., 1997). The Pennsylvania State University extension publication focus entirely on the ventilation and the designs that facilitate appropriate and necessary ventilation (Fabian, 2002). Finally, the *Horse Facilities Handbook* by Eileen Wheeler is also a more comprehensive discussion of equine facilities which includes arenas. The common theme among the mentioned publications is the lack of information about indoor arenas. The two publications that discuss them, *Horse Handling Facilities* and *Horse Facilities Handbook*, briefly mention them and provide cursory recommendations about dimension, construction materials, and suggest using principles like the ones used to build outdoor arenas, but that indoor arenas may experience issues due to too little ventilation (Kidd et al., 1997; Wheeler, 2005).

In 2014 and 2015, the International Federation for Equestrian Sports (Fédération Équestre Internationale or FEI) published the Equestrian Surfaces – A Guide and the Equine Surfaces White Paper, respectively. Both publications focus on important considerations for arena footing, such as the biomechanics of footfall of the horses, the differences in footing types, and how the usage of the arena impacts all aspects of the arena design and building process (Lonnell and Hernlund, 2014; Hobbs et al., 2015). The FEI’s publications provide information regarding how to construct an arena with the focus being on the sub-base, base, and footing surface or cushioning, but does reiterate the statement by Wheeler et al. that an indoor arena vs. an outdoor will have different management issues (Wheeler, 2005; Lonnell and Hernlund, 2014; Hobbs et al., 2015). The *Equine Surfaces White Paper* recognizes that many environmental factors must be taken into consideration, such as the potential issues due to the presence of organic materials and the creation of dust from the footing, and acknowledges that more research is necessary to fully understand the potential impacts. Spending substantial amounts of time within indoor arenas has been linked to higher occurrences of self-reported respiratory conditions in equine instructors (Kollar et al., 2005). The percentage of instructors who reported incidence of bronchitis symptoms was higher for those who were identified as indoor instructors compared to those who reported to be smokers (Kollar et al., 2005).

The connection between the exposure to inhalable dust and immune response has also been documented in the literature. Endotoxins and β(1→3) glucans have been reported within the horse stables in amounts higher than normal recommended exposure levels, but little research has been conducted on the exposure within indoor arenas (Elfman et al., 2009; Samadi et al., 2009). In addition, the increase in ventilation rates within stables has been shown to decrease the inflammatory markers present within horse stable workers in conjunction with a reduction in overall allergens, such as ammonia and ultrafine particles (Wålinder et al., 2011). Kentucky Equine Research published a short article detailing that arena dust may have an impact on the lung health of horses (Kentucky Equine Research Staff, 2017). A study conducted in Germany examining the changes in dust dispersed in indoor arenas with the addition of horses working in the arena determined that, overall, the addition of a working horse substantially increased the amount of dust present in the indoor arena environment (Lühe et al., 2017). The research conducted thus far states that dust within horse facilities is a potential hazard to human and horse health and indicates a need for further research into the impacts of dust and other exposures within indoor arenas.

There has been limited research and publications regarding indoor arenas, their design and construction, and the environment other than studies on dust production and moisture content with regards to the footing (Lühe et al., 2017; Claußen et al., 2019). The survey designed and distributed was intended to help fill in the gaps in the research and to begin to identify concerns and gather basic information from the facility owners, managers, and riders who used them. The main objectives of the survey were to begin to characterize building dimensions, footing, and indoor climate, define typical maintenance procedures and practices, and explore the potential health issues created in these facilities due to dust, moisture, and ventilation design.

## METHODS AND MATERIALS

### Study Design

In order to best capture the varied experiences of those in the horse industry, the online survey was designed to have different questions for people who owned, managed, or rode regularly at facilities with indoor arenas. The survey was completely voluntary, and the respondents could opt out of the survey at any time. If the participant was under 18 or did not ride in an indoor arena on a regular basis, they were unable to participate.

The survey was constructed using the Qualtrics survey software that is used for all official University of Kentucky surveys and was approved under Internal Review Board (IRB) protocol number 44274. Using this software allowed for the questions to be presented to the participant based off the answers to the initial questions, such as do you own, manage, or ride in a facility with an indoor arena. Respondents who selected that they owned or managed a facility with an indoor arena were also asked if they rode in one. If they selected that they both owned and managed a facility with an indoor arena, they were only asked the questions the owners were asked as they were similar question sets, but more information was asked of the owners. For instance, riders were asked questions regarding their riding habits and general questions about the farm where they ride, the arena design and size, the footing, and the arena environment. Managers answered similar questions to the riders, but also included questions about the arena construction, its orientation, eave and ridge height, arena maintenance and the farm occupancy. Finally, the owners were asked questions regarding the cost to build and operate the indoor arena in addition to all the questions the riders and managers were asked. All three of the groups were also asked a series of health-related questions to begin to identify any health concerns for the horses and humans that use the arena.

Distribution of the survey was done through multiple equine news outlets and organizations. The organizations shared a link to the survey on their websites, in their publications, and on their social media pages depending on how they reach their members. The goal with the selection of the various organizations and media outlets was to reach as many people as possible from a multitude of different equine disciplines. The organizations can be found in Table 1. There were other organizations and individuals that chose to share the survey on their own, but the ones who wrote letters of intent that were filed on the IRB are listed.

**Table 1.** The organizations listed on the IRB that shared the survey

United States EquestrianFederation (USEF)	Mid-south eventing and dressage association
Paulick Report	Eventing Nation
Jumper Nation	American Horse Publications
UK Equine Programs	Bluegrass Equine Digest
Appaloosa Horse Club	Kentucky Horse Council

The survey was open for a 12-wk period from the beginning of May 2018 to the end of July 2018. In this time, 455 people took the survey from 9 different countries. Of the 455 respondents, 339 results were used for the analysis as they had completed 80% or more of the survey. Demographics for the survey respondents are included in Table 2. The survey consisted of 154 questions, though some questions were specifically for owners, managers, or riders, so no one answered all the questions. Questions were either multiple choice, select all that apply, or open-ended questions with some of the multiple-choice questions having an option for a text answer. All analysis was completed on the aggregate data and no one individual or facility could be identified from the survey questions and results. The goal of the study is to characterize and identify issues within indoor arenas not to damage the reputation of any facility or individual.

**Table 2.** Demographics of the survey respondents

Age *N* = 352	18–24	20.7%
	25–34	29%
	35–44	18.1%
	45–54	13.6%
	55–64	12.8%
	65+	5.1%
	Choose not to answer	0.5%
Gender *N* = 352	Male	4.3%
	Female	95.4%
	Choose not to answer	0.3%
Race/ethnicity *N* = 353	American Indian or Alaska Native	0.6%
	Asian	0.9%
	Black or African American	0.6%
	Hispanic	0.6%
	Other	0.6%
	White	93.5%
	Choose not to answer	3.4%
Own, manage or ride in a facility with an indoor arena *N* = 353	Own	13.3%
	Manage	10.2%
	Ride	76.5%

### Statistical Analysis

Analysis of the data was completed using the JMP software. Based off the topics and questions asked of the respondents, relationships from the data were determined. The queries of the survey data were designed to examine the effect of characteristics such as age, region, cost to build the arena, and environmental concerns on traits such as lighting, footing type, number of windows, size of the arena, and the definition of an indoor arena.

Due to the large and varied distributions of the some of the answers, it was necessary to recode responses. For instance, the question regarding which state the respondents owned, managed, or rode in an indoor arena were recoded into regions of the United States. The regions used were the Northeast, South, Midwest, Kentucky, Southwest, West, and outside the United States. Kentucky was left as its own region as it had the largest number of respondents and was the location where site visits planned for future research. Other questions recoded included the disciplines in which the respondents rode, arena construction materials, arena size, footing contents, environmental concerns, and footing treatment methods. All the recoding information can be found in Supplementary Appendix A.

Main discipline of the farms where the originally categorized as Dressage, Show Jumping/Hunter Jumper, Eventing, Reining, All Around, Gymkhana/Games, Gaited, Endurance, Racing, Fox hunting, Driving, Western Performance, and Other, which the respondent was asked to select all that applied. The disciplines were recoded to All Around, Dressage, Eventing, Flat and Fence Emphasis, and Flat Emphasis. Respondents who selected only All Around, Dressage, or Eventing were kept in those categories; if only one discipline was selected that was not one of those three the responses were recoded to Flat and Fence Emphasis or Flat Emphasis depending on whether jumping was part of the selected response. Finally, if multiple disciplines were selected, they were recoded into the Flat and Fence Emphasis group or the Flat Emphasis group according to whether a jumping discipline was one of the selected disciplines.

The most notable recoding was for the footing within the indoor arenas. The footing was divided into two different categories: the primary component and any secondary components found in the footing. Primary components included sand, waxed sand, washed sand, crushed rock, dirt, dirt and sand and wood chip, while the secondary components were clay, crushed rock, fiber, fiber and rubber, none, rubber, and wood chip. Crushed rock and wood chip were included in both categories because there were arenas that indicated the only footing found was crushed rock or wood chip, but there were also arenas which indicated it was included with other footing types such as sand or dirt.

Once the recoding was complete, contingency tables examining the relationships between answers to different questions were completed. By examining the relationships between the answers, trends present within the data can be explored within the different categories. For example, the relationship between the age of an indoor arena and the type of lighting present could be examined to determine whether the age of the arena had any effect on the lighting used in the facility. As all the data was categorical, all plots created were mosaic plots. *P*-values were included in the contingency tables and *P*-values less than 0.05 were considered significant, while *P*-values between 0.1 and 0.05 were considered trending towards significant.

## RESULTS AND DISCUSSION

### Age of Arena

**Figure 1.**
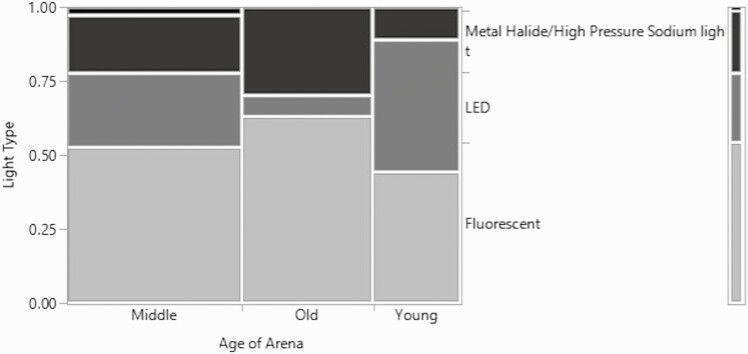
Mosaic plot for the relationship between the age of the arena and the lighting type.

Only the owners and managers reported the age of the arena, so the responses only included the answers from those two respondent groups. Age of arena was evaluated for its relationship to the lighting, the primary component of the footing, any secondary components of the footing, and the square footage or size of the arena. By combining the answers of the owners and managers, the sample size averaged 83 answers for the questions regarding the age of the arena. A possible trend between age of arena and lighting was identified (Figure 1); the *P*-value for the likelihood ratio of the relationship between age and the lighting within the indoor arena was 0.08 (*χ*^2^ = 11, *n* = 81, df = 6). Metal halide/high pressure sodium lights and fluorescent lights were most prevalent in arenas older than 5 yr. Most interestingly, LED lights were seen most in the 1–5 yr old arenas and the arenas 6–15 yr old. One reason for this trend in arenas older than 15 yr is that the older arenas are being retrofitted with newer, more energy efficient lights as the metal halide, high pressure sodium or fluorescent lights failed or became too difficult to maintain. While LED lights are more expensive to install than fluorescent lights, many farms consider them to be more economical considering maintenance and operating costs.

**Figure 2.**
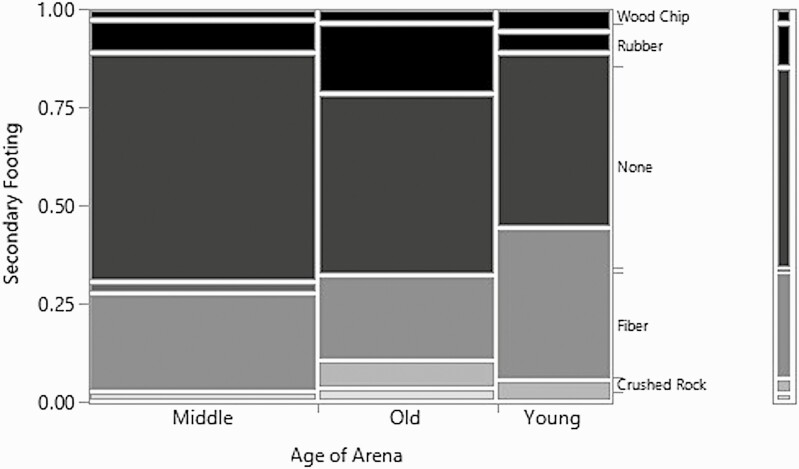
Mosaic plot for the relationship between the age of the arena and the secondary footing component.

Examining the primary and secondary footing components did not yield any significant relationship between the age of the indoor arena and the footing (*χ*^2^ = 15.9, *P*-value = 0.19, *n* = 82, df = 12 and *χ*^2^ = 10.1, *P*-value = 0.6, *n* = 82, df = 12, respectively). The only notable trend was the primary component of the footing which showed that no arenas under 5 yr old contained any primary footing component other than sand, while the arenas that were 6–15 yr or older than 15 yr had more variability in the primary footing component. The most common secondary footing type was none meaning that there wasn’t anything added to the primary footing component while the second most common secondary footing component was fiber (Figure 2). The arenas under 5 yr old did have a higher amount of fiber as a secondary footing component than the 6–15 yr old or older than 15 yr arenas.

**Figure 3.**
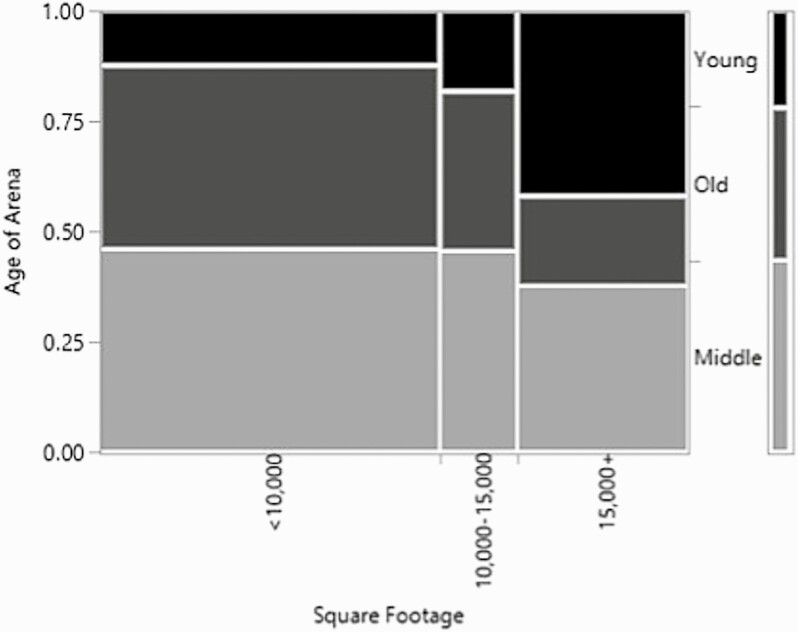
Mosaic plot for the relationship between the age of the arena and the square footage of the indoor arena.

Finally, the relationship with the age of the arena and the square footage of the arena was examined (Figure 3). The square footage was broken down in less than 10,000 ft^2^, 10,000–15,000 ft^2^, and over 15,000 ft^2^. Similar to the relationship with the lighting, the relationship between the age of the arena and the size of the arena were trending towards significance (*χ*^2^ = 8.2, *P*-value = 0.08, *n* = 83, df = 4). The arenas that were over 5 yr old were more likely to be less than 10,000 ft^2^, the arenas between 6 and 15 yr old were more likely to be 10,000–15,000 ft^2^ or less than 10,000 ft^2^, and then the newer arenas, those under 5 yr old, were more likely to be over 15,000 ft^2^. This indicates a trend that the more recently the arena was built, the larger it tends to be.

### Cost to Build

**Figure 4.**
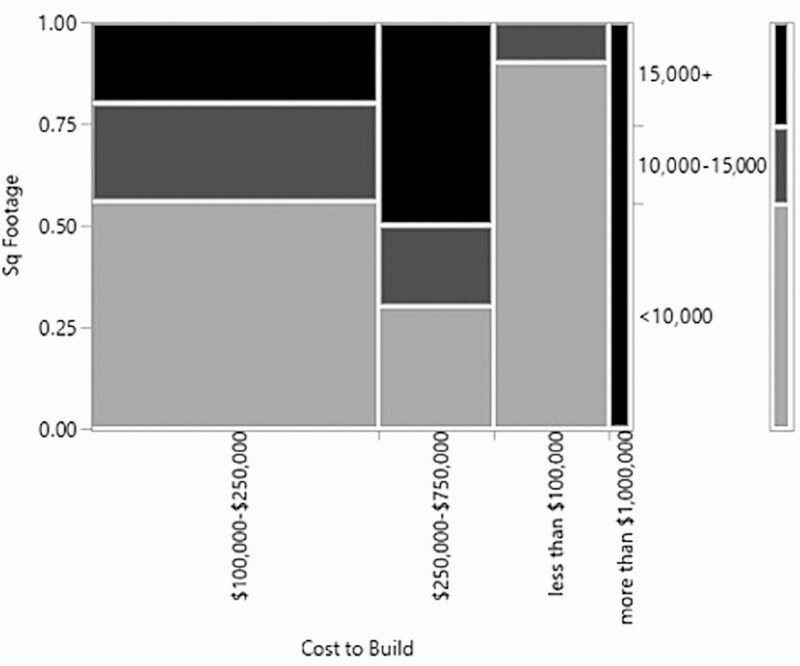
Mosaic plot of the relationship between the cost to build the arena and the square footage of the arena.

Unlike the questions regarding the age of the arena, only the owners were asked the cost to build the arena and this led to a reduction in the number of responses regarding cost, as only 47 of the respondents were owners of facilities with an indoor arena. Interestingly, the cost to build the arena did not have a significant relationship with the lighting type, either the primary or secondary footing component, or the main discipline of the farm. This is potentially due a lack of information because of the relatively small sample size or because there are no trends associated with the cost of the arena and the examined arena traits. As expected though, the square footage of the arena and the cost to build the arena was a significant relationship (*χ*^2^ = 16.8, *P*-value = 0.01, *n* = 47, df = 6) (Figure 4). The most expensive arenas, which were over $1,000,000, were also some of the largest. However, there were arenas that were over 15,000 ft^2^ that also cost between $100,000 and $250,000 to build. This indicates that the most expensive arenas incurred costs in other areas such as being wider rather than longer, using more expensive building materials, having the ability to heat or cool the space, or something else entirely. The most significant trend that was seen is that the largest percentage of the arenas built were less than 10,000 ft^2^ and cost less than $250,000 to build.

### Region

Responses for all three groups, owners, managers, and riders, were combined to examine relationship with the region where their indoor arena was located. The number of lights in the indoor arena and the primary footing component were not significant, but also didn’t display any interesting or notable tendencies with the data. However, the relationship between the region and the secondary footing component was highly significant (*χ*^2^ = 85.4, *P*-value < 0.001, *n* = 324, df = 36) (Figure 6). The most prevalent answer was no secondary components in Kentucky, the Northeast, and outside the United States. In comparison, fiber was the most common secondary component in the South.

**Figure 5.**
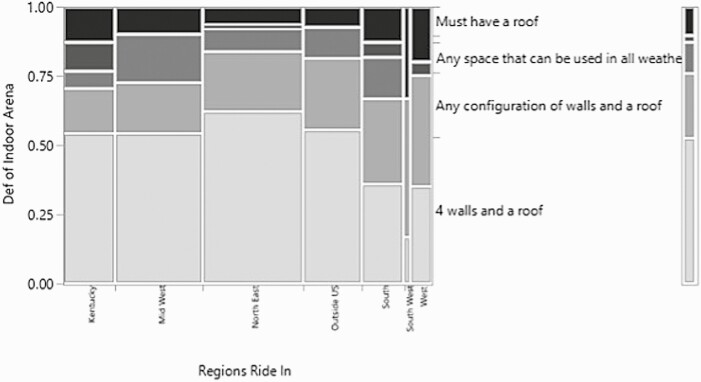
Mosaic plot of the relationship between the region respondents ride in/where the arena is located and how respondents define an indoor arena.

**Figure 6.**
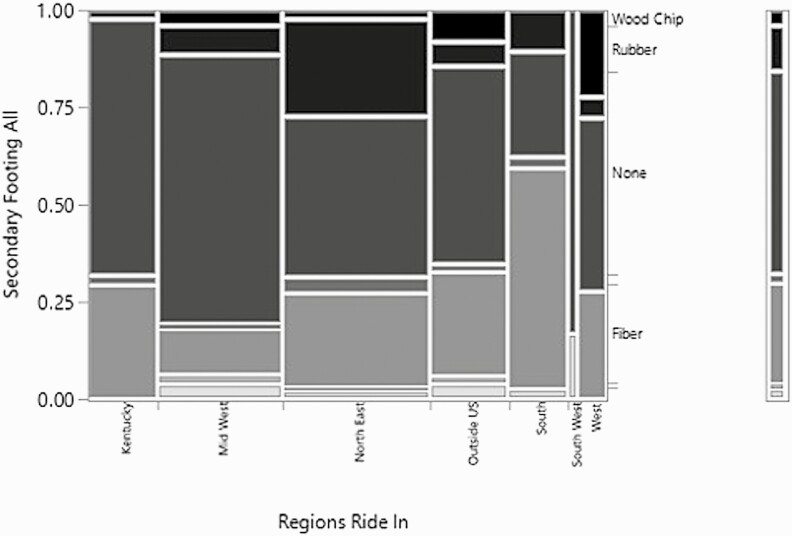
Mosaic plot of the relationship between the region respondents ride in/where the arena is located and the secondary footing component.

There was a significant relationship for the regions where the indoor arenas were located and how the respondents of the survey defined an indoor arena (*χ*^2^ = 45.9, *P*-value = 0.0045, *n* = 341, df = 24) (Figure 5). For the majority of respondents in Kentucky, the Northeast, the Midwest, and outside the U.S. regions, the definition of an indoor arena was “four walls and a roof’, but for the South, Southwest, and West, four walls and a roof was closely matched by or less than ‘any configuration of walls and a roof.’’ Overall, the third most common answer was any space that can be used in all weather. The division between the more northern states and the southern states is quite striking for this question. Areas of the United States which experience colder and more inclement weather tended towards expecting four walls and a roof to be considered an indoor arena, while the regions that require more air movement due to hotter conditions or which receive less inclement weather, indicated an arena was any configuration of walls and a roof. This seems to indicate that the different regions have different requirements for their indoor arenas and, therefore, have different overall ideas of how an indoor arena appears.

**Figure 7.**
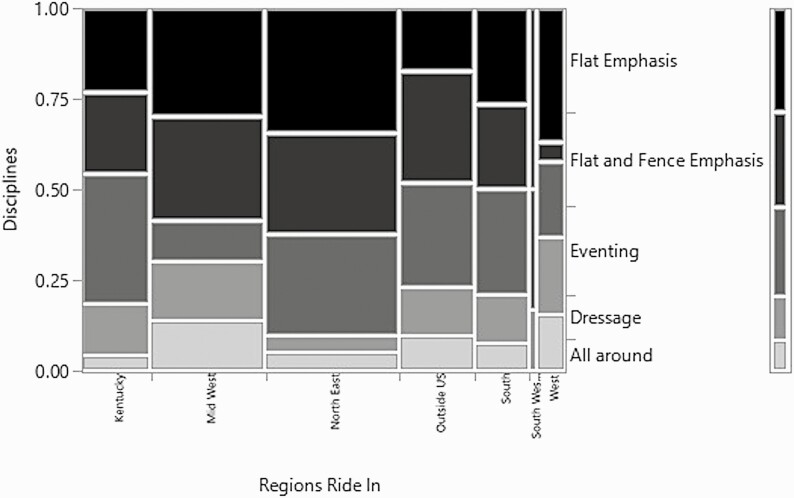
Mosaic plot of the regions respondents ride in/where the arena is located and the discipline the respondents ride.

Examining regions and the main riding discipline of the farms (*χ*^2^ = 40.3, *P*-value = 0.01, *n* = 336, df = 24) yielded more regional differences (Figure 7). Dressage was most prevalent in the Midwest region, but the highest number of farms were classified as a flat emphasis and a fence and flat emphasis. Kentucky had the highest percentage of eventing farms over the other disciplines. The Northeast, the South, and outside the US contained the highest numbers of eventing farms, but both the South and the Northeast had an even distribution of eventing farms, flat emphasis, and flat and fence emphasis. Outside of the US only differed in that flat emphasis was not as large a percentage of the disciplines reported. Little research has been completed regarding regional differences and riding disciplines and this indicates more research in this area is warranted.

When coupled with the significance of the relationship between the regions and the main disciplines of the farms, this trend of secondary footing components is compelling. Fiber as a secondary footing component is more prevalent in the South and Northeast and the disciplines that tend to use fiber in their footing are more prevalent in those regions of the United States as well. Examining the relationship between the discipline and the secondary footing indicates that disciplines with a jumping or jumping and flat emphasis have a higher prevalence of fiber in the footing. Fiber is added to footing to increase shear strength, stability, and to reduce maintenance requirements while increasing the grip of the footing in combination with a sand footing (Lonnell and Hernlund, 2014). The addition of fiber is a common occurrence for the sport horse disciplines (show jumping, dressage, and eventing), but the effect on the horses has not been greatly studied (Lonnell and Hernlund, 2014).

While the relationship between the region and the square footage of the arenas was not significant, there was a trend in the data (*χ*^2^ = 17.5, *P*-value = 0.1313, *n* = 322, df = 12). Overall, the arenas tended to be either less than 10,000 ft^2^ or more than 15,000 ft^2^. There were some arenas that were in the range from 10,000 to 15,000 ft^2^, but it was a much smaller percentage. In addition to the square footage relationship, whether the indoor arena was attached to the barn was not significant though Kentucky and the Midwest trended towards more facilities being attached, while the Northeast trended towards more facilities not being attached. Arenas and barns were considered attached if the horses could walk from one location to the other without going outside. Both of these relationships warrant more research to see if there are regional design preferences that could be established with larger sample sizes.

**Figure 8.**
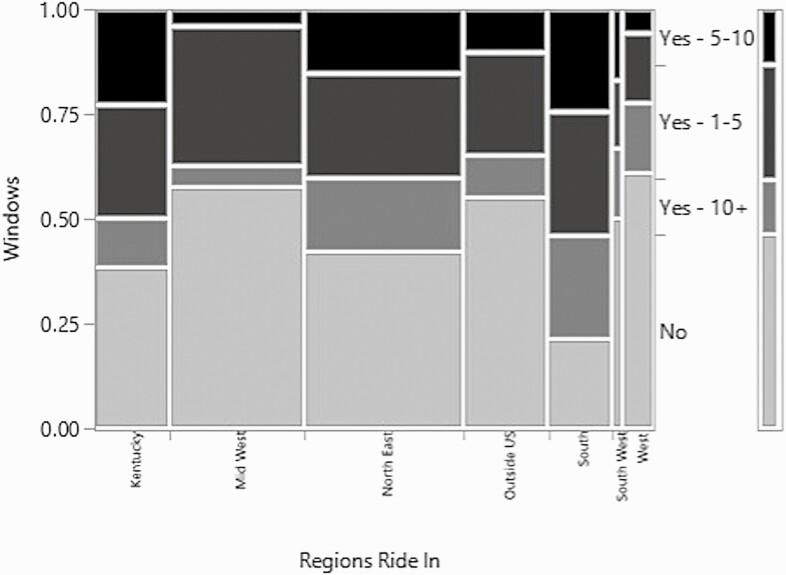
Mosaic plot of the relationship between the number of windows in the indoor arena and the regions the respondents ride in/where the indoor arena is located.

The number of windows in the facility was also examined in relation to the region the arena was located and there was a significant association between the two (*χ*^2^ = 36.8, *P*-value = 0.005, *n* = 324, df = 18) (Figure 8). Facilities in Kentucky, the Northeast, the Midwest, the West, and outside the US all most frequently reported not having windows. Having fewer than five windows was the second most frequent answer for all the afore mentioned regions except for the West. The South tended to have windows, but the number of windows was evenly distributed between less than 5 windows, 5–10 windows, and more than 10 windows. Considering that the weather in the South tends to be hotter and more humid than other regions, windows that can facilitate air movement may be more regularly included in indoor arena designs.

### Environmental Concerns

Of the 335 respondents of the survey, 71.3% or 239 respondents reported having concerns about the environment within the indoor arena. The respondents could select all the environmental concerns that they had for their arena so someone could have selected dust, moisture, and lack of air. Due to this, the frequencies were calculated based on each concern (Table 3). Dust is anecdotally discussed in literature with a few specific studies and therefore expected to be a problem within indoor arenas but having 84.5% of respondents who identified environmental concerns choose dust strongly reinforced that dust was an issue. In addition to dust being a concern, lack of air movement was a concern for 65.3% of those who had concerns regarding the environment. It is possible that both concerns are because of a lack of appropriate ventilation within the facility. By changing the indoor arena’s ventilation system, it might be possible to help eliminate some of these concerns with dust and a lack of air movement. These changes may be as simple as having appropriate inlets and outlets to move air through the indoor arena. One other possibility for these issues is due to arena maintenance practices. Moisture was a concern for 27.2% of respondents in the indoor arena they owned, managed, or rode in. The most common method for controlling dust is by distributing water on the footing, which may create condensation challenges due to too much moisture or even challenges getting adequate moisture applied. Many of these environmental concerns may be interrelated and could be mitigated through similar methods. For instance, increasing the numbers of windows, doors, and translucent panels in the indoor arena, could mitigate the concerns regarding lack of air movement, temperature issues, and not having enough light within the arena. In addition, it is possible that while a solution may mitigate one concern it could exacerbate another. Many arenas treat footing with water in order to keep dust down; applying additional water to reduce dust could potentially cause problems with increased moisture in the arena environment. Ultimately, managing environmental concerns will require additional studies in order to balance addressing one problem without creating another.

**Table 3.** Percentage of respondents who identified environmental concerns within the indoor arenas. The percentages are based on the number of respondents who reported having at least one environmental concern

Environmental concern	Percent
Dust	84.5
Moisture	27.2
Lack of air movement	65.3
Too much air movement	1.67
Not enough light	25.1
Temperature	36.8

### Discipline Specific Needs

**Figure 9.**
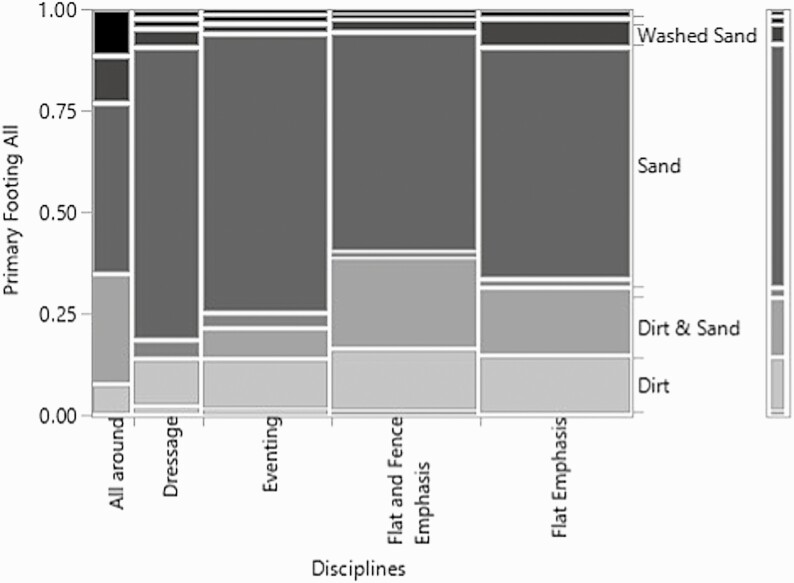
Mosaic plot of the relationship the discipline respondents rode and the primary footing component.

The relationship between the different disciplines and the primary footing component was significant (*χ*^2^ = 49.7, *P*-value = 0.007, *n* = 336, df = 28) (Figure 9). Regardless of discipline, sand was the most common primary footing component. An interesting trend within the fence and flat emphasis and the flat emphasis was the second most common primary footing component was a dirt and sand mixture especially as many dirt surfaces are common for racetracks and in the racing industry, while when mixing with fiber which is common in the sport horse disciplines, sand without dirt or woodchips mixed in is advised (Lonnell and Hernlund, 2014; Hobbs et al., 2015). In contrast, the secondary component of the footing and its relationship with discipline was only trending towards significance (*χ*^2^= 33.3, *P*-value = 0.09, *n* = 336, df = 24). Once again, the most prevalent answer was not having any secondary components in the footing. Fiber was the second most prevalent answer of possible secondary footing components with dressage, eventing, flat emphasis, and flat and fence emphasis being the disciplines that reported more than 20% of the arenas of that discipline type containing fiber. This trend is consistent with the previous survey data regarding the disciplines and footing components that was observed examination of the relationship between regions and disciplines and footing components.

The square footage or size of the arena was not significantly correlated with the different disciplines (*χ*^2^ = 4.0, *P*-value = 0.85, *n* = 334, df = 8). While there were not any trends in the data with respect to disciplines, the majority of the indoor arenas split between being less than 10,000 ft^2^ and more than 15,000 ft^2^. This split has been seen in other relationships where arena size has been examined such as in the discussion regarding the regions where the indoor arenas were located.

### Building Dimensions

**Figure 10.**
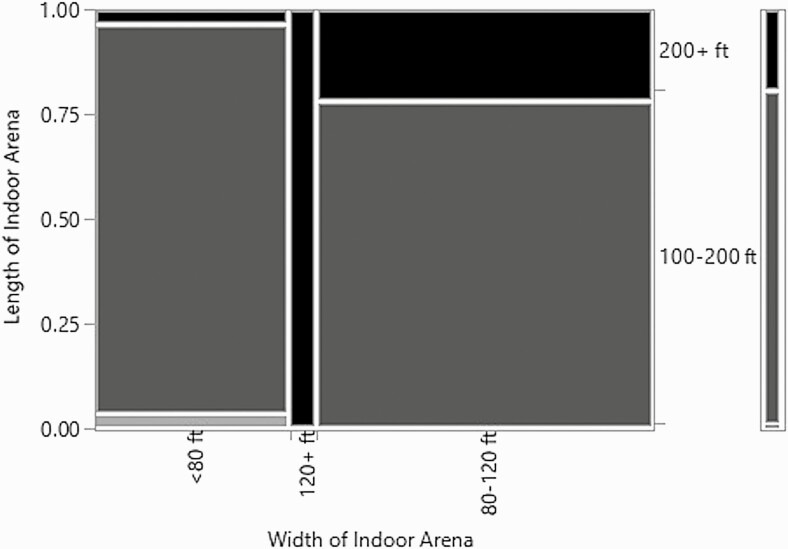
Mosaic plot of the relationship between the length of the arena and the width of the arena.

The relationship between the length and the width of the arenas was significant (*χ*^2^ = 21.7, *P*-value = 0.0002, *n* = 82, df = 4) (Figure 10). Most of the data were concentrated into the mid-range of length and width: between 100–200 ft long and 80–120 ft wide. Understandably, none of the arenas less than 100 ft long were over 80 ft wide. Any arenas that were wider than 120 ft were also longer than 200 ft. These relationships are expected with the general rule of thumb that arenas are longer than they are wide. Comparing width and length with the eave height did not result in any significant relationships.

## CONCLUSION

Indoor arenas allow trainers and riders to work and train their horses no matter the season or the weather outside. They serve an important purpose within the industry that relies heavily on consistency of being able to train so the horses can compete and, therefore, generate profits for their owners and trainers. The survey indicates trends and changes that have been occurring within the industry which is vital when advising farms as they build new indoor arenas or retrofitting existing facilities. The use of LED lights and constructing larger arenas are some of the most notable trends across the entire United States which would directly impact designers and builders of indoor arenas. One of the main indications from the survey is that respondents consider indoor arenas to be facilities that have four walls and a roof or any configuration of walls and a roof. While this is the main definition of indoor arenas, this definition is highly dependent on the region where the indoor arena is located. In addition, there is a clear objective to address environmental concerns within these facilities as 79% of respondents stated they had concerns with the environments in indoor arenas. These concerns highlight the potential for health issues for the humans and horses using the facilities and the construction and maintenance of the facilities need to be adjusted to address them. By directly addressing the notable trends or areas of change within indoor arena, structures newly built or retrofitted can make the best use of monetary investments and set farms up for future success and business and facility longevity.

## Supplementary Material

txab198_suppl_Supplementary_Appendix
