# Supplementary material for: Characterization of indoor arenas through an anonymous survey
Source: Transl Anim Sci. 2021 Oct 9;5(4):txab198. doi: 10.1093/tas/txab198 (PMC8576445; doi:10.1093/tas/txab198)
Supplement: txab198_suppl_Supplementary_Appendix [file txab198_suppl_supplementary_appendix.docx]

# Appendix 1 – Survey Recoding

| **Original Question** | **Original Answers** | **Recoded Answers** |
| --- | --- | --- |
| In which state do you currently own a facility, manage a facility, or ride at a facility with an indoor arena? | All 50 states and Outside the US | Kentucky, Northeast, South, Southwest, West, Midwest, and Outside the US |
| Select the discipline(s) that best describe the main riding discipline on your farm or you participate in: | Dressage, Show Jumping/Hunter Jumper, Eventing, Reining, All around, Gymkhana/Games, Gaited, Endurance, Racing, Fox hunting, Driving, Western performance, Other (please specify) | All Around, Dressage, Eventing, Flat and Fence Emphasis, Flat Emphasis |
| What was the cost to build your indoor arena facility? | Less than $100,000, $100,000-$250,000, $250,000-$500,000, $500,000-$750,000, $750,000- $1,000,000, more than $1,000,000 | Less than $100,000, $100,000-$250,000, $250,000-$750,000, more than $1,000,000 |
| How old is the indoor arena? | Less than 5 years old, 5-10 years old, 10-15 years old, 15-20 years old, more than 20 years old | 1-5 years old, 6-15 years old, 15+ years old |
| What most accurately represents the dimensions of your arena? (width x length) | 60 ft x 100 ft, 80 ft x 120 ft, 100 ft x 200 ft, 150 ft x 300 ft, Other (please specify) | Width: less than 80 ft, 80 – 120 ft, more than 120 ft |
|  |  | Length: less than 100 ft, 100-200 ft, more than 200 ft |
|  |  | Square Footage: less than 10,000 sq ft, 10,000-15,000 sq ft, more than 15,000 sq ft |
| What type of lighting do you have? | Fluorescent linear/bar light; Fluorescent high bay, Fluorescent round light, LED linear/bar light, LED round light, Metal halide/high pressure sodium light, Other (Please specify) | Fluorescent, LED, Metal halide/high pressure sodium light, Spotlight |
| Are horses stabled under the same roof as the arena? | No, Yes - horses are in the same space as the arena, Yes – a door can be closed, Yes- doorway is open constantly | No, Yes |
| What best describes the footing in the indoor arena? (Select all that apply) | Dirt, Sand, Washed Sand, Waxed Sand, Poly/Synthetic, Clay, Crushed Rock, Wood Chip/Sawdust, Textile/fiber – low proportion, Textile/fiber – high proportion, Rubber, Other (Please specify) | Primary Footing Component: Crushed Rock, Dirt and Sand, Sand, Washed Sand, Waxed Sand, Wood Chip |
|  |  | Secondary Footing Component: Clay, Crushed Rock, Fiber, Fiber and Rubber, None, Rubber, Wood Chip |
